# Regulation of Fatty Acid Oxidation in Mouse Cumulus-Oocyte Complexes during Maturation and Modulation by PPAR Agonists

**DOI:** 10.1371/journal.pone.0087327

**Published:** 2014-02-05

**Authors:** Kylie R. Dunning, Marie R. Anastasi, Voueleng J. Zhang, Darryl L. Russell, Rebecca L. Robker

**Affiliations:** School of Paediatrics and Reproductive Health, The Robinson Institute, The University of Adelaide, Adelaide, South Australia, Australia; Konkuk University, Republic of Korea

## Abstract

Fatty acid oxidation is an important energy source for the oocyte; however, little is known about how this metabolic pathway is regulated in cumulus-oocyte complexes. Analysis of genes involved in fatty acid oxidation showed that many are regulated by the luteinizing hormone surge during in vivo maturation, including acyl-CoA synthetases, carnitine transporters, acyl-CoA dehydrogenases and acetyl-CoA transferase, but that many are dysregulated when cumulus-oocyte complexes are matured under in vitro maturation conditions using follicle stimulating hormone and epidermal growth factor. Fatty acid oxidation, measured as production of ^3^H_2_O from [^3^H]palmitic acid, occurs in mouse cumulus-oocyte complexes in response to the luteinizing hormone surge but is significantly reduced in cumulus-oocyte complexes matured in vitro. Thus we sought to determine whether fatty acid oxidation in cumulus-oocyte complexes could be modulated during in vitro maturation by lipid metabolism regulators, namely peroxisome proliferator activated receptor (PPAR) agonists bezafibrate and rosiglitazone. Bezafibrate showed no effect with increasing dose, while rosiglitazone dose dependently inhibited fatty acid oxidation in cumulus-oocyte complexes during in vitro maturation. To determine the impact of rosiglitazone on oocyte developmental competence, cumulus-oocyte complexes were treated with rosiglitazone during in vitro maturation and gene expression, oocyte mitochondrial activity and embryo development following in vitro fertilization were assessed. Rosiglitazone restored *Acsl1*, *Cpt1b* and *Acaa2* levels in cumulus-oocyte complexes and increased oocyte mitochondrial membrane potential yet resulted in significantly fewer embryos reaching the morula and hatching blastocyst stages. Thus fatty acid oxidation is increased in cumulus-oocyte complexes matured in vivo and deficient during in vitro maturation, a known model of poor oocyte quality. That rosiglitazone further decreased fatty acid oxidation during in vitro maturation and resulted in poor embryo development points to the developmental importance of fatty acid oxidation and the need for it to be optimized during in vitro maturation to improve this reproductive technology.

## Introduction

Oocytes acquire their developmental competence, the ability to undergo successful fertilization and development into an embryo, during ovarian folliculogenesis. Ovarian follicle growth begins from the primordial stage where a small oocyte is surrounded by a single layer of somatic cells known as granulosa cells. These proliferate and differentiate until the preovulatory stage where a fully grown oocyte is surrounded by specialized cumulus cells, a fluid filled antral cavity and a stratified epithelial layer of granulosa cells. The final stages of oocyte developmental competence are acquired following a surge of luteinizing hormone (LH) from the pituitary which signals to the preovulatory follicle, via the granulosa cells, to ovulate. During this time maturation of the oocyte resumes and includes meiotic progression to metaphase II in preparation for fertilization in the oviduct.

The in vitro maturation (IVM) of oocytes involves the isolation of an immature oocyte and companion cumulus cells, known collectively as the cumulus oocyte complex (COC), prior to the LH-surge, followed by hormone treatment in vitro [1], [2]. Thus, IVM occurs in the absence of the normal follicular environment resulting in numerous deficiencies, including altered energy metabolism, compared to in vivo matured COCs [3]–[5]. Oocytes generated by IVM have poorer development following fertilization and result in higher miscarriage rates compared to in vivo matured oocytes [6]–[8]. Thus IVM is infrequently used in clinical practice due to the poor quality of oocytes generated using this reproductive technology. The mechanisms underlying the poor quality following IVM are not evident; however it is understood that cellular metabolism and metabolic rate of the oocyte and cumulus cells are a determinant of oocyte quality [9]–[13] with ATP levels within the oocyte positively correlated with developmental potential [14].

Lipids are metabolized for the generation of ATP by the process of fatty acid oxidation (FAO), which is emerging as an important process in oocyte meiotic maturation [15], [16] and early embryo development [17]–[19]. In fact there has been much interest in up-regulating FAO during IVM to improve oocyte quality [17], [18], [20]–[24]. Further, inhibition of FAO during IVM is associated with poor embryo development [17], [25]. Thus, FAO plays an important role in oocyte developmental competence, yet the normal in vivo regulation of this metabolic pathway during COC maturation has not been described. Further, whether COCs matured in vitro achieve equivalent levels of FAO is not known.

Fatty acid oxidation can be modulated in numerous tissues, via activation of peroxisome proliferator activated receptor (PPAR) signalling pathways. PPARs are nuclear receptor transcription factors that regulate the metabolism of lipids [26]–[28] and there are three major types, PPARα [29], PPARγ and PPARδ [30], each of which are endogenously activated by prostaglandins and fatty acids [31]. Fibrates such as bezafibrate and thiazolidinediones such rosiglitazone are potent pharmaceutical activators of PPARα and PPARγ, respectively [32]. We have previously shown that in vivo administration of rosiglitazone in the periovulatory period was able to reverse the negative effects of obesity and a high fat diet on oocyte developmental competence in the mouse [33]. Yet whether PPAR agonists directly affect FAO in the COC or alter developmental competence is unknown.

Thus in the current study we sought to determine whether the expression of genes involved in the FAO pathway are regulated in the COC during in vivo maturation and whether similar levels are achieved during IVM. We also determine whether FAO is deficient during IVM using a functional assay and investigate the effect of PPAR agonists on FAO and oocyte developmental competence.

## Materials and Methods

### Ethics Statement

All experiments were approved by the University of Adelaide's Animal Ethics Committee (approval number M-2009-164) and conducted in accordance with the Australian Code of Practice for the Care and Use of Animals for Scientific Purposes.

### Materials

Equine chorionic gonadotropin (eCG) and follicle stimulating hormone (FSH) were purchased from the National Hormone and Peptide Program (NHPP) (Tovance, CA, USA). Human chorionic gonadotropin (hCG; Pregnyl) was purchased from Lyppard Australia Pty. Ltd. (Keysborough VIC). Culture medium was purchased from Gibco, Invitrogen Australia Pty. Ltd. All other reagents were purchased from Sigma-Aldrich Pty. Ltd. unless otherwise indicated.

### Animals

All mice were purchased from Laboratory Animal Services (Adelaide, SA) and were maintained on a 12L:12D photoperiod with rodent chow and water provided ad libitum.

### Isolation of Mouse COCs

Prepubertal (day 21–23 of age) CBA × C57BL/6 first filial generation (F1) (CBAF1) female mice were treated with intraperitoneal administration of eCG (5IU) alone or followed by intraperitoneal administration of hCG (5 IU) 44–48 h later. Ovaries were dissected from mice and placed in Hepes-buffered minimum essential medium alpha (αMEM) containing 5% fetal calf serum (FCS). At 46 h post eCG (immature; 0 h hCG) or 6, 10 or 16 h following administration of hCG (preovulatory; in vivo matured), COCs were collected by puncture of large antral follicles, or the oviduct (16 h post-hCG) with a 30-gauge needle to release COCs. Approximately 30 COCs are obtained from each mouse. For the in vitro maturation of COCs, immature COCs isolated from ovaries of at least 6 mice at 46 h post-eCG were pooled and matured for 10 or 14 h in bicarbonate buffered αMEM supplemented with 5% FCS, 5 ng/ml epidermal growth factor (EGF), 50 mIU/ml FSH, and 0.25 mM pyruvate at 37°C and 6% CO_2_.

### Gene Expression Analysis

In three independent experiments, COCs were isolated from 12 mice, pooled and frozen in liquid nitrogen. RNA was extracted from COCs using the RNeasy Mini Kit (Qiagen, VIC, Australia) as per the manufacturer's instructions. RNA concentration and purity were quantified using a Nanodrop ND-1000 Spectrophotometer (Biolab Ltd., Victoria, Australia). Reverse transcription to cDNA was performed using 236, 300 or 114 ng of RNA (COC in vivo maturation time course; in vivo versus IVM COC expression; and effect of rosiglitazone on gene expression during IVM, experiments respectively) and the RT^2^ First strand kit (Qiagen, VIC, Australia) as per manufacturer's instructions, which includes a genomic DNA elimination step. Analysis of gene expression was performed using a 384-well Fatty Acid Metabolism RT^2^ Profiler Array (SA Biosciences, Qiagen, VIC, Australia). PCR cycling conditions were 95°C for 10 min, followed by 40 amplification cycles of 95°C for 15 seconds and 60°C for 1 min. The geometric mean of at least two genes, as indicated in figure legends, were used for normalization of data following assessment of reference gene stability using BestKeeper [34]. Data were analyzed using the 2^−(ΔΔCT)^ method.

### β-Oxidation Assay

Fatty acid oxidation of 9,10-[^3^H]palmitate substrate was determined by measuring the production of ^3^H_2_O over 4 hours in COCs matured by IVM or in vivo for 10 h, as described [17]. COCs were isolated from 10 mice, pooled and added at 40 per well to each well of a 96-well plate containing bicarbonate buffered αMEM supplemented with 0.3 mM 9,10-[^3^H]palmitate (32.4 Ci/mmol), 5% FCS, 0.25 mM pyruvate, 50 mIU/ml FSH, 5 ng/ml EGF, 3 mM D-glucose and 0.25 mM L-carnitine in a final volume of 100 µl for 4 h at 37°C/6% CO_2_.

To determine the effect of PPAR agonists bezafibrate and rosiglitazone on β-oxidation in the COC during IVM, β-oxidation was measured during COC maturation over 20 h. The PPARα agonist, bezafibrate, was used at 0, 50, 100, 250, 500, and 800 μM (diluent: ≤0.01% DMSO) and the PPARγ agonist, rosiglitazone at 0, 0.5, 1, 5, 20, 50 or 100 μM (diluent: ≤0.01% DMSO) based on studies in other cell types [35]–[40]. Twenty immature unexpanded COCs (44–48 h eCG) were added to a well of a 96-well plate containing bicarbonate buffered αMEM supplemented with 0.3 mM 9,10-[^3^H]palmitate (32.4 Ci/mmol), 5% FCS, 0.25 mM pyruvate, 50 mIU/mlFSH, 5 ng/ml EGF, 3 mM/L D-glucose and 0.25 mM L-carnitine and the required dose of agonist, in a final volume of 100 µl, and incubated for 20 h at 37°C and 6% CO_2_.

For β-oxidation assays each treatment was performed in either triplicate or duplicate and duplicate blank wells containing no COCs included for baseline subtraction. Excess 9,10-[^3^H]palmitate was precipitated as described in [17]. The aqueous phase (0.5 ml) was combined with 1 ml scintillation fluid and counted on a Beckman LS 6000LL β-counter for 20 minutes. β-oxidation levels were calculated by first subtracting the mean of the duplicate blank wells, then dividing the ratio of labelled product (Ci) to labelled substrate (32.4 Ci/mmol), and then calculating the proportion of tritiated to unlabeled hydrogen atoms. All β-oxidation data are presented as pmol palmitic acid/COC/hour. Calculations were based on the assumptions of no loss of tritiated water through evaporation and complete oxidation of the radiolabeled substrate.

### Assessment of Cumulus Expansion

Cumulus expansion was assessed following 14 h of IVM by an independent assessor blinded to all treatments. The established Vanderhyden scale was used to score the COC expansion from 0–4 [41], with 0 indicating no expansion and 4 expansion of all layers of cumulus cells.

### Analysis of Mitochondrial Activity

Mitochondrial membrane potential in oocytes from COCs matured in vitro for 14 h in the presence or absence of rosiglitazone (20 µM) was determined using JC-1 (5,5′,6,6′-tetrachloro-1,1′,3,3′-tetraethylbenzimidazolylcarbocyanine iodide; Invitrogen). COCs were collected following 14 h of IVM in bicarbonate buffered αMEM supplemented with 5% FCS, 5 ng/ml EGF, 50 mIU/ml FSH, 0.25 mM pyruvate. Oocytes were denuded using hyaluronidase (100 IU/ml) and manual pipetting with a fine bore pipette, then stained in 6 µM JC-1 dissolved in Hepes buffered αMEM/0.2% polyvinylpyrrolidone (PVP) for 15 mins at 37°C. Oocytes were then mounted in 3 µl between 2 glass coverslips and separated by a spacer (Invitrogen, secure-seal spacer, one well 13 mm diameter, 0.12 mm deep) and imaged immediately in both green and red fluorescence channels (Fluoview, FV10i Olympus confocal microscope). The plane of focus in which the oocyte diameter was largest was assumed to be the center and was selected for image capture and analysis.

Fluorescence intensity was quantified using analySIS pro software (Olympus Australia Pty. Ltd., Mt Waverly, VIC, AU). A region of interest (ROI), of fixed size that encompassed the entire oocyte diameter was used to provide an average intensity of fluorescence within the oocyte. An identical ROI was used for each oocyte in all experiments.

### In Vitro Fertilization and Embryo Development Assessment

COCs were collected following 14 h of IVM in bicarbonate buffered αMEM supplemented with 5% FCS, 0.25 mM pyruvate, 50 mIU/ml FSH, 5 ng/ml EGF, 3 mM D-glucose, 0.25 mM L-carnitine and with or without rosiglitazone (20 µM). Sperm were collected from CBA × C57BL/6 F_1_ male mice from the vas deferens and the caudal region of the epididymis and capacitated in bicarbonate-buffered αMEM supplemented with 3 mg/ml BSA (fatty acid free) for 1 h at 37°C in an atmosphere of 6% CO_2_ and 94% air. Following capacitation, COCs and sperm were co-incubated in bicarbonate-buffered αMEM supplemented with 3 mg/ml BSA for 4 h at 37°C in an atmosphere of 6% CO_2_ and 94% air. Presumptive zygotes were stripped of any remaining cumulus cells and sperm by manual pipetting and cultured in 20 µl drops of “Vitro Cleave” (A905969; COOK Australia, Brisbane, QLD, Australia) overlaid with mineral oil. Presumptive zygotes were cultured at 37°C in an atmosphere of 6% CO_2_ and 94% air. Fertilization rate was scored 24 hours post-insemination (day 2) based on the division of the oocyte into a two-cell embryo. On day three of embryo culture (48 h post-insemination), the embryos were moved to a new 20 μl culture drop of “vitro cleave” medium (A905969, Cook Medical). Embryo development was assessed on day 3 (48 h post-insemination), 4 (78 h post-insemination) and day 5 (96 h post-insemination) of embryo culture for development to the 4–8 cell, ≥ morula and blastocyst stages of development, respectively.

### Statistical Analyses

Results are presented as the mean ± SEM. Statistical analyses were performed as indicated in figure legends using GraphPad Prism Version 5.1 for Windows (GraphPad Software, Inc.). All data were checked for normality and transformed where necessary as indicated in the figure legends. One-way ANOVA, two-way ANOVA, and *t* tests were used as described in the figure legends and statistical significance was considered at a P value of <0.05. Proportional data (embryo development) were arcsine transformed prior to statistical analysis.

## Results

### Temporal Expression of Genes Involved in Fatty Acid Oxidation during Oocyte Maturation In Vivo

**Figure 1:**
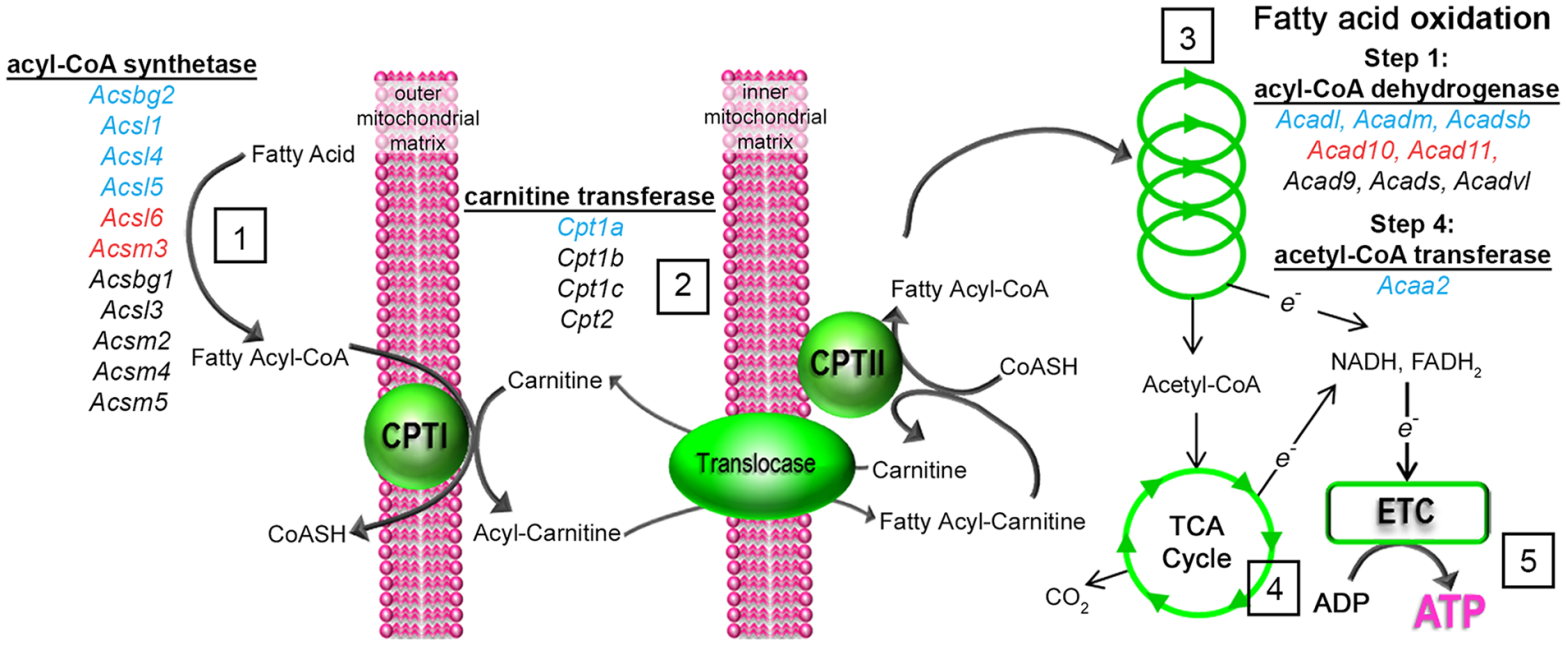
Long chain fatty acid activation, entry into mitochondria and metabolism via fatty acid oxidation. 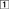
 Entry of long chain fatty acids into mitochondria requires activation by acyl-CoA synthetase enzymes which catalyze the transfer of CoA from CoA-SH to form fatty acyl-CoA. 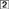 Activated fatty acids enter mitochondria via enzymatic transfer of CoA for carnitine which is catalyzed by carnitine palmitoyl transferase I (CPTI). Fatty acyl-carnitine enters the mitochondrial matrix via carnitine acylcarnitine translocase where carnitine palmitoyl transferase II (CPTII) replaces carnitine with CoA. This is known as the carnitine shuttle. 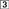 Fatty acyl-CoA then enters the fatty acid oxidation spiral which has 4 steps catalyzed by 1) fatty acyl CoA dehydrogenase, 2) enoyl CoA hydratase, 3) hydroxyacyl CoA dehydrogenase and 4) acetyl-CoA transferase (also known as ketoacyl-CoA thiolase) and yields an acetyl-CoA molecule for each cycle. 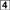 Acetyl-CoA is able to enter the tricarboxylic acid (TCA) cycle which with fatty acid oxidation generates electrons forming NADH and FADH_2_ which donate electrons to the electron transport chain (ETC) required for ATP synthesis 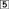 Genes involved in fatty acid oxidation were measured in cumulus oocyte complexes during in vivo maturation in response to hCG. A summary of their expression pattern is depicted with blue, red and black coloured genes representing significantly up-regulated, down-regulated and unchanged respectively, as demonstrated in Figure 2.

**Figure 2:**
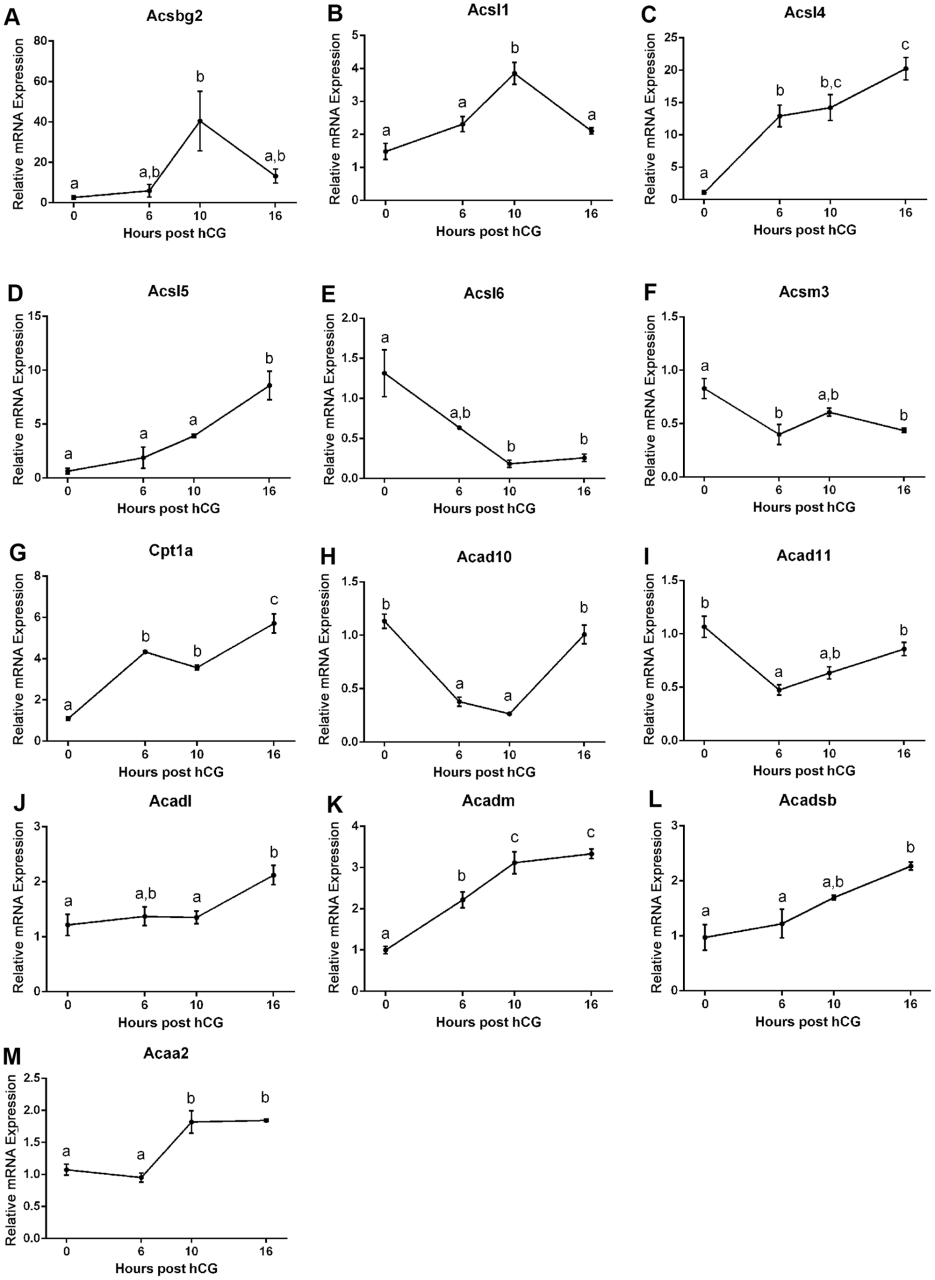
Modulation of genes involved in fatty acid oxidation in the cumulus oocyte complex during oocyte maturation in vivo. Analysis of *Acsbg2*, *Acsl1*, *Acsl4*, *Acsl5*, *Acsl6*, *Acsm3*, *Cpt1a*, *Acad10*, *Acad11*, *Acadl*, *Acadm*, *Acadsb*, and *Acaa2* (**A–M**, respectively) expression in cumulus oocyte complexes at 0, 6, 10, and 16 h post-hCG administration is shown. mRNA expression was normalized to the geometric mean of *Gusb*, *Hprt*, *Actb*, *Gapdh* and *Hsp90ab1* and presented as mean ± SEM (n = 3 experimental replicates, different superscripts signify statistical difference of *P*<0.05 by one-way ANOVA with Tukey post hoc test).

We first determined whether genes involved in the fatty acid oxidation (FAO) metabolic pathway (Fig. 1) are regulated in the COC following an ovulatory dose of hCG to induce oocyte maturation in vivo. We analysed the expression of several isoforms of acyl-CoA synthetases, which activate long chain fatty acids by catalyzing their conversion to acyl CoA derivatives allowing transport into mitochondria, and found that *Acsbg2*, *Acsl1*, *Acsl4* and *Acsl5* were significantly induced during oocyte maturation in vivo while *Acsl6* and *Acsm3* were significantly down-regulated (Fig. 2A–F). The expression of *Acsbg1*, *Acsl3*, *Acsm2*, *Acsm4* and *Acsm5* were not regulated during in vivo maturation (data not shown). Expression of carnitine transporters *Cpt1a*, *Cpt1b*, *Cpt1c* and *Cpt2* which transport activated long chain fatty acids into the mitochondria revealed that *Cpt1a* was significantly induced (Fig. 2G) while the other genes remained unaltered (data not shown).

Analysis of acyl-CoA dehydrogenase isoforms, which catalyze the first step in the FAO spiral, revealed that *Acad10* and *Acad11* were significantly altered post hCG demonstrating an initial down regulation at 6 h post hCG while at 16 h, post ovulation, expression was not significantly different from immature 0 h COCs (Fig. 2H and I). *Acadl*, *Acadm* and *Acadsb* were significantly induced in the COC during maturation in vivo (Fig. 2 J–L). The fourth and final step in the FAO spiral is performed by acetyl-CoA transferase and encoded by *Acaa2*. *Acaa2* expression was significantly up-regulated during oocyte maturation in vivo compared to immature 0 h COCs (Fig. 2M). These results show that a number of FAO genes are dynamically regulated in COCs in vivo in response to ovulatory hCG and that even distinct isoforms of similar enzymes exhibit differential expression patterns (see Fig. 1 for summary of changes).

### Fatty Acid Oxidation Pathway Genes are Dysregulated in COCs Matured In Vitro

**Figure 3:**
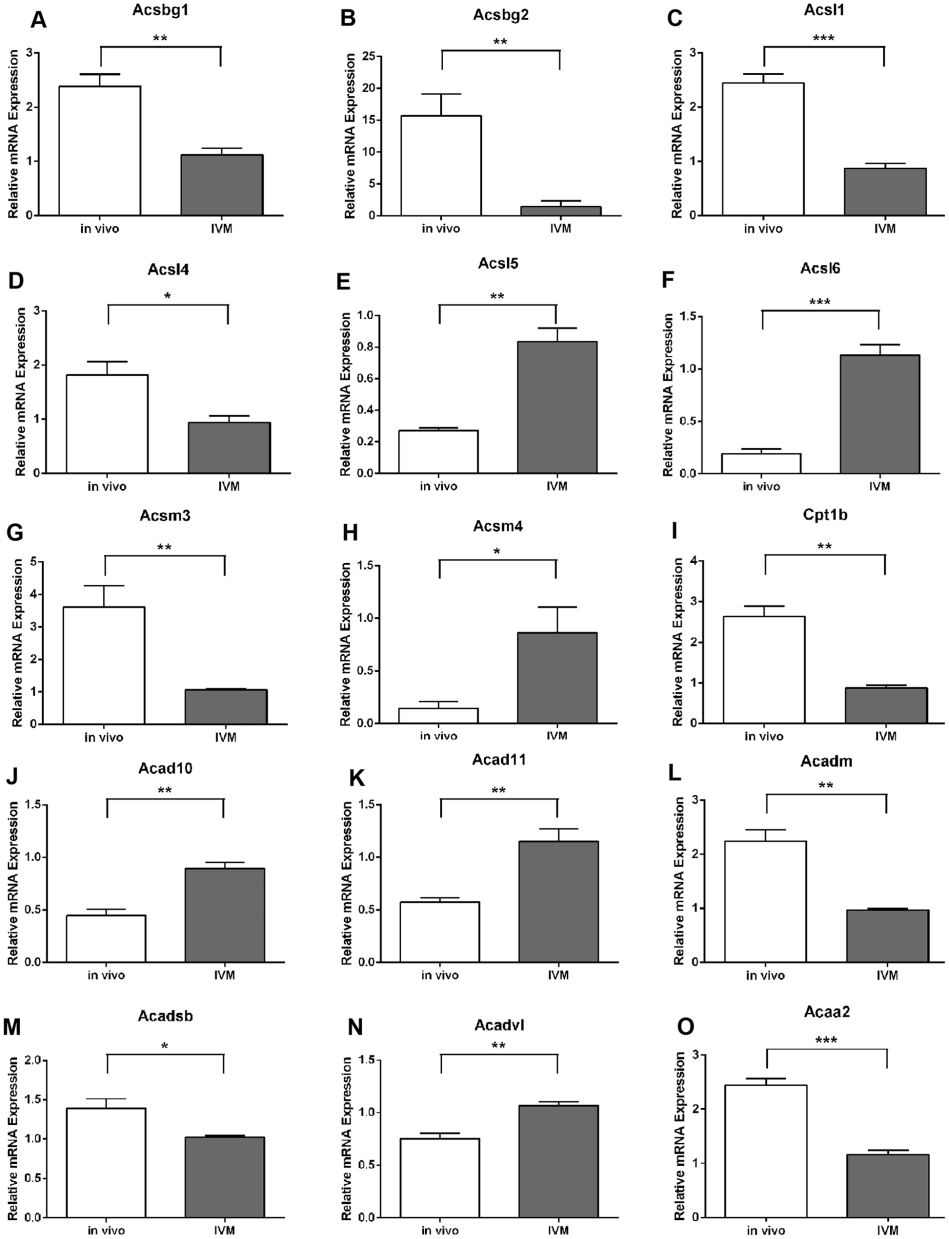
Gene transcripts involved in fatty acid oxidation are dysregulated in cumulus oocyte complexes matured in vitro (IVM). Analysis of *Ascbg1*, *Acsbg2*, *Acsl1*, *Acsl4*, *Acsl5*, *Acsl6*, *Acsm3*, *Acsm4*, *Cpt1b*, *Acad10*, *Acad11*, *Acadm*, *Acadsb*, *Acadvl*, and *Acaa2* (**A**-**O**, respectively) expression in cumulus oocyte complexes following 10 h of in vivo or in vitro maturation (IVM). mRNA expression was normalized to the geometric mean of *Gapdh* and *Hsp90ab1* and presented as mean ± SEM (n = 3 experimental replicates, * *P*<0.05, ** *P*<0.01, *** *P*<0.001 by unpaired t test).

We next assessed whether COCs matured in vitro had altered expression of these dynamically regulated fatty acid oxidation genes. The time point of 10 h maturation was chosen for comparison since the majority of genes were significantly different at this time point in vivo compared to levels at 0 h (Fig. 2). There was no difference in cell number between COCs from the two maturation conditions (in vivo: 3040±205.5; IVM: 2457±377.3 cells/COC). Analysis of COCs matured for 10 h in vitro showed that Acyl-CoA synthetase expression was significantly altered in IVM COCs compared to in vivo matured COCs with *Acsbg1*, *Acsbg2*, *Acsl1*, *Acsl4* and *Acsm3* expression significantly decreased in IVM COCs at 10 h while *Acsl5*, *Acsl6* and *Acsm4* were significantly higher compared to in vivo matured COCs (Fig. 3 A–H). *Acsl3*, *Acsm2*, and *Acsm3* were not different in COCs matured in vivo for 10 h compared to non-matured COCs (Fig. 2 and data not shown) nor compared to COCs matured in vitro (data not shown). Expression of the isoform *Cpt1b* was significantly reduced in IVM COCs (Fig. 3I) while *Cpt1a*, *Cpt1c* and *Cpt2* were unaltered (data not shown). Genes encoding isoforms for acyl-CoA dehydrogenases which perform the first step in the FAO spiral showed disparate expression patterns with *Acad10*, *Acad11* and *Acadvl* significantly higher in IVM COCs while *Acadm* and *Acadsb* were significantly reduced compared to in vivo matured COCs (Fig. 3 J–N). *Acadl*, *Acad9* and *Acads* expression levels were not different between maturation treatment groups (data not shown). The expression of the acetyl-CoA transferase *Acaa2*, which catalyzes the final step in the FAO spiral, showed a significant decrease following IVM (Fig. 3O).

**Figure 4:**
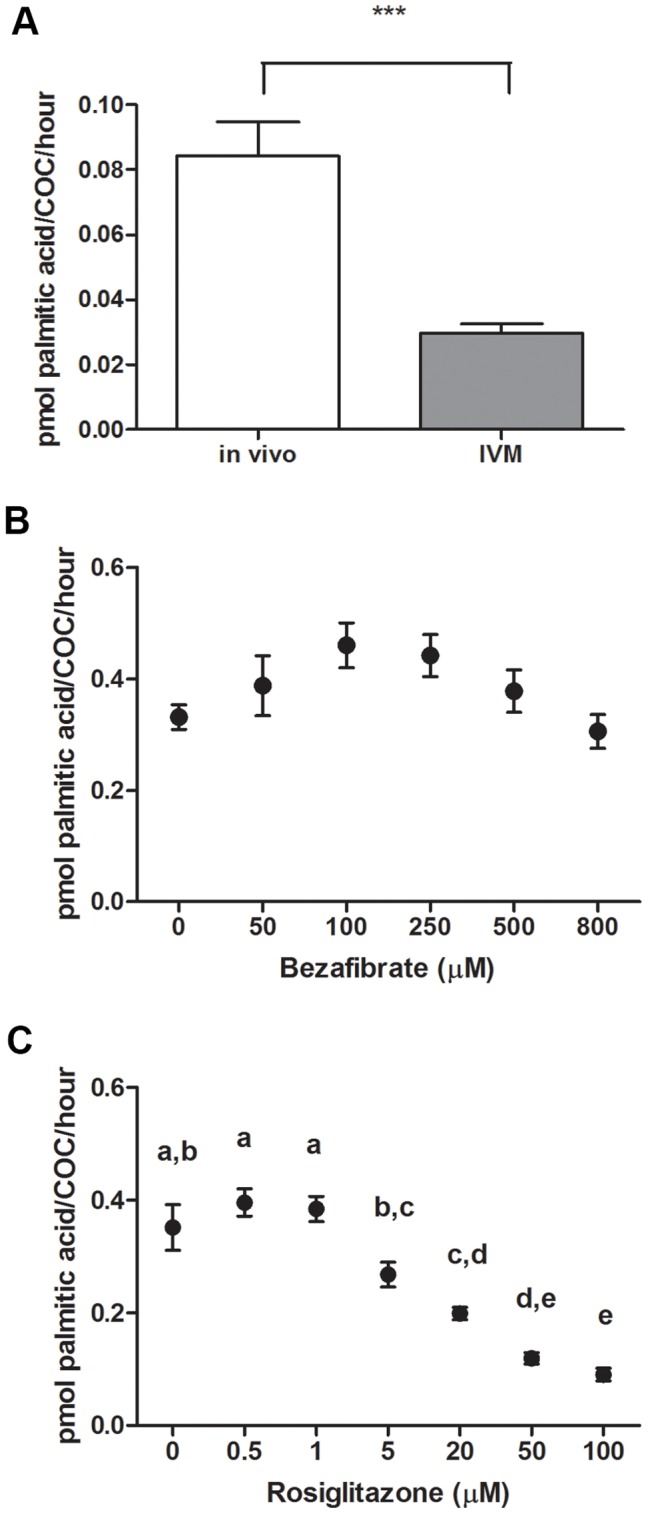
FAO is reduced in cumulus oocyte complexes matured in vitro and is modulated by rosiglitazone but not bezafibrate. Cumulus oocytes complexes (COC) were matured for 10 h in vitro (IVM) or collected 10 h following the administration of hCG (in vivo matured). β-oxidation was measured over 4 h of culture and expressed as pmol palmitic acid metabolized per COC per hour (**A**). Data presented as mean ± SEM, n = 7 per treatment from 3 independent experiments, representative of 280 COCs per treatment. ***P<0.001 by unpaired *t* test. The effect of PPAR agonists bezafibrate and rosiglitazone on β-oxidation was measured in COCs maturing in vitro over 20 h in the presence of increasing doses of bezafibrate (**B**) or rosiglitazone (**C**). Data presented as mean ± SEM, n = 5 independent experiments, representative of 100 COCs per group. Data analyzed by one-way ANOVA and Tukey post hoc test, different letters signifying statistical difference (*P*<0.05).

We next compared functional rates of FAO in COCs that were matured in vitro for 10 h versus COCs isolated from follicles at 10 h post-hCG (i.e. matured in vivo). There was a significant 2.8-fold reduction in the level of FAO occurring in COCs matured in vitro (IVM) compared to those matured in vivo (Fig. 4A). Thus, IVM conditions result in COCs performing significantly less FAO than in vivo matured COCs at this timepoint.

### Effect of PPAR Agonists on COCs during In Vitro Maturation

Because of the dramatically reduced level of FAO in IVM COCs, we next determined whether agonists of PPARs, specifically bezafibrate and rosiglitazone, would increase FAO in COCs during in vitro maturation. Real-time RT-PCR was used to confirm the presence of *Pparα*, *Pparg* and *Ppard* mRNA transcripts in COCs prior to experiments examining the effects of the PPAR agonists (data not shown). Treatment of COCs with bezafibrate using doses ranging from 50–800 µM had no significant effect on FAO rate during the maturation period (Fig. 4B). At 100 µM bezafibrate did result in a 1.4-fold increase in FAO compared to control, however this dose also failed to have any significant effect over control conditions in subsequent assays investigating oocyte quality (data not shown). Conversely, treatment of COCs with rosiglitazone during IVM significantly reduced FAO in COCs in a dose dependant manner (Fig. 4C).

**Figure 5:**
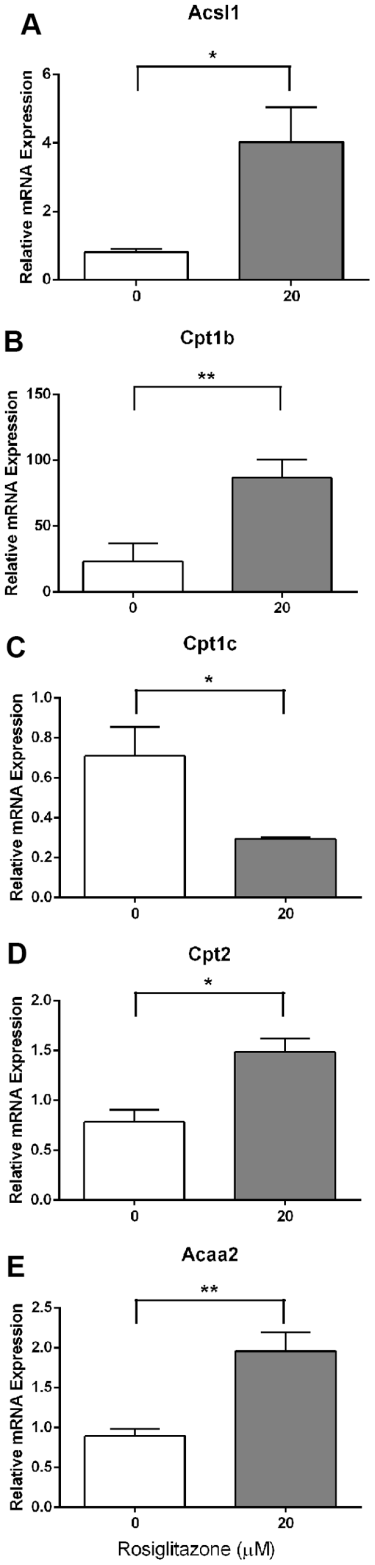
Rosiglitazone treatment of COCs during in vitro maturation significantly modulates expression of genes involved in fatty acid oxidation. Analysis of *Acsl1* (**A**), *Cpt1b* (**B**), *Cpt1c* (**C**), *Cpt2* (**D**) and *Acaa2* (**E**) expression in cumulus-oocyte complexes following 10 h of in vitro maturation (IVM) in the absence (0) or presence of rosiglitazone (20 µM). mRNA expression was normalized to the geometric mean of *Gapdh* and *Hsp90ab1* and presented as mean ± SEM (n = 3 experimental replicates, * *P*<0.05, ** *P*<0.01 by unpaired t test).

Interestingly, despite rosiglitazone treatment (20 µM) significantly inhibiting FAO in COCs during IVM this dose caused a significant increase in the expression of genes involved in fatty acid activation (*Acsl1*), carnitine mediated transport (*Cpt1b* and *Cpt2*) and the FAO spiral (*Acaa2*) compared to untreated controls (Fig. 5). In contrast, expression of *Cpt1c* was significantly decreased in the presence of rosiglitazone (Fig. 5C). The remaining 19 genes measured during in vivo maturation described above (or see Fig. 1) were also assessed but did not show significant modulation by rosiglitazone. Rosiglitazone treatment (20 µM) also significantly increased the degree of cumulus expansion compared to control (cumulus expansion index: control: 3.42±0.05 vs. rosiglitazone: 3.58±0.05, *P*<0.05, Mann Whitney *t* test due to non-normal distribution of data, n = 7 experimental replicates, representative of ≥155 COCs per treatment).

These results show that rosiglitazone significantly inhibits FAO in the COC during IVM despite upregulating a subset of genes involved in the FAO metabolic pathway.

### Effect of Rosiglitazone on Oocyte Quality

**Figure 6:**
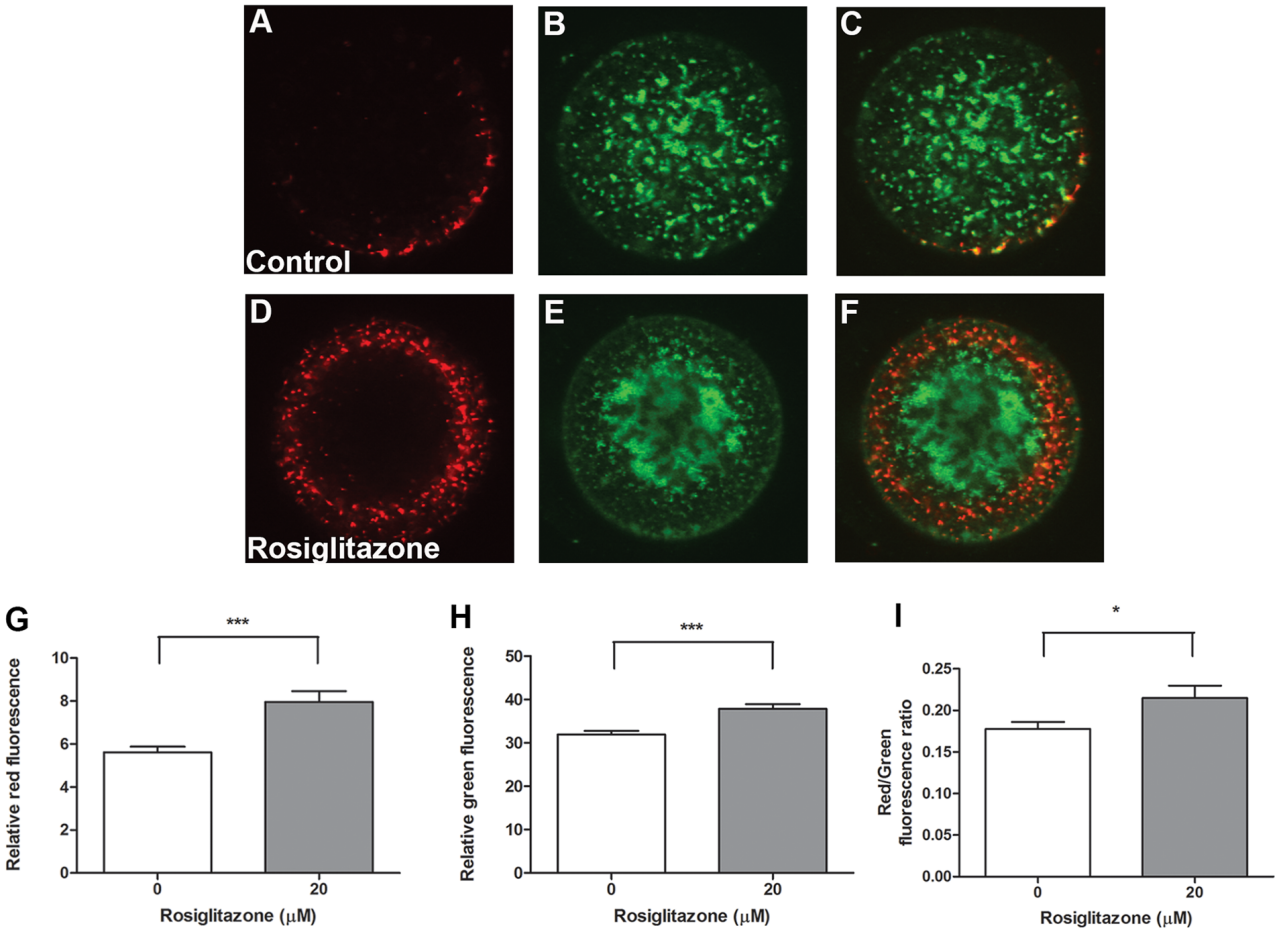
Mitochondrial membrane potential is increased in oocytes following maturation of cumulus oocyte complexes in vitro in the presence of rosiglitazone. Mitochondrial membrane potential was assessed by JC-1 staining of oocytes following in vitro maturation of cumulus oocyte complexes in the absence (**A–C**) or presence of 20 µM rosiglitazone (**D–F**). Red (**G**), green (**H**) and the ratio of red:green (**I**) fluorescence within oocytes was quantified and presented as mean ± SEM, representative of 30–35 COCs per group, from 3 independent experiments. Data analyzed by unpaired *t* test on untransformed (**H**) or Log10 transformed (**G** and **I**) data, * *P*<0.05; *** *P*<0.001.

Oocyte mitochondrial membrane potential analyzed by JC-1 staining was altered by rosiglitazone (20 µM) during in vitro maturation of COCs (Fig. 6 A–F). Quantification of fluorescence confirmed that rosiglitazone treatment of COCs resulted in oocytes with a higher red:green fluorescence ratio indicating increased mitochondrial activity and that this was due to significantly higher levels of both red and green fluorescence(Fig. 6 G–I). Further, the distribution of green fluorescence appeared altered in rosiglitazone treated COCs with more of these mitochondria localised in the center of the oocyte (Fig. 6E) compared to a more homogeneous distribution observed in the oocytes matured in control conditions (Fig. 6B).

**Figure 7:**
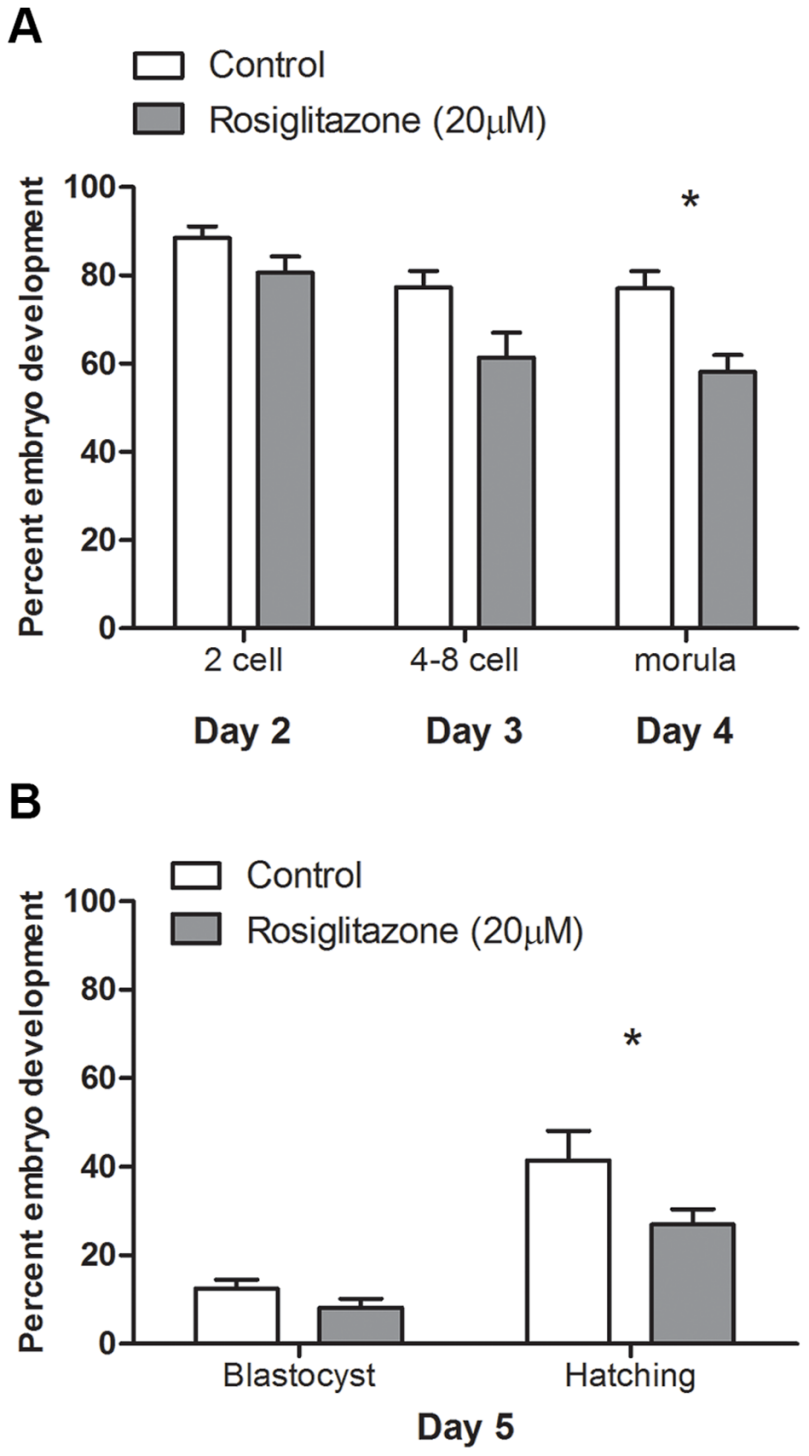
Rosiglitazone treatment during in vitro maturation of cumulus-oocyte complexes negatively affects oocyte developmental competence. Oocyte developmental competence was assessed following in vitro fertilization of cumulus-oocyte complexes matured in vitro in control conditions or in the presence of rosiglitazone (20 µM). Embryo development was assessed on days, 2, 3, 4 and 5, with day 1 designated as the day of fertilization (**A** and **B**). Development on day 5 was assessed as blastocyst or hatching blastocyst (**B**). Data presented as mean ± SEM, n = 7 independent experiments, representative of 132–138 oocytes per treatment group. Arcsine transformed data were analyzed by either repeated measures two-way ANOVA (**A**) or regular two-way ANOVA with Bonferroni post hoc test (**B**). Asterix indicates statistical difference (*P*<0.05) within developmental stage.

COCs matured in vitro in the presence of rosiglitazone (20 µM) did not differ significantly in their ability to be fertilized and develop into a 2-cell embryo compared to COCs matured under control conditions (Fig. 7A). However, rosiglitazone treated COCs resulted in significantly fewer morula embryos on day 4 of embryo culture (Fig. 7A) and significantly fewer hatching blastocyst embryos on day 5 compared to control (Fig. 7B).

Together these data show that despite rosiglitazone treatment of COCs increasing oocyte mitochondrial membrane potential, indicative of mitochondrial hyperpolarization, this was associated with decreased oocyte developmental competence with fewer fertilized oocytes capable of developing to the blastocyst stage of preimplantation development compared to oocytes from COCs matured in control conditions.

## Discussion

Little is known about the regulation of FAO in the COC during maturation and how it impacts subsequent embryo development. In the current study we have shown that genes involved in FAO are hormonally regulated in the COC during maturation in vivo and are dysregulated during in vitro maturation. Since mature oocytes are transcriptionally inactive and oocyte mRNA would represent a small fraction of the mRNA in the COC, these dynamic changes in expression levels are attributed to changes in cumulus cells. This supports previous reports demonstrating that β-oxidation by the oocyte represents a small fraction of that occurring in the whole COC [16], [17]. Further, using a functional assay we show that FAO is decreased in COCs matured in vitro compared to those that mature in vivo within the follicular environment. Whether decreased FAO persists throughout maturation in vitro remains to be determined, as does whether this reduction is directly due to the identified alterations in gene expression or deficiencies in other factors, such as growth factors and specific metabolites that are lacking in in vitro systems. Lastly, we show that treatment with the PPARγ agonist, rosiglitazone, significantly inhibits FAO and results in poor developmental competence.

The significant upregulation of acyl-CoA synthetases *Acsbg2*, *Acsl1*, *Acsl4*, and *Acsl5* in the COC during maturation in vivo indicate that the COC is preferentially utilizing fatty acids with chain lengths of C10-C20 [42]–[45]. This correlates well with the predominant forms of fatty acids present in human and bovine follicular fluid; palmitic, oleic, linoleic and arachidonic acid [46]–[49] for which Acsl1, Acsl5 and Acsl4 have high affinity [43]–[45]. Significant 5-fold induction of *Cpt1a* during maturation in vivo indicates that there is increased capacity for carnitine-mediated transport of fatty acids into mitochondria which is required for the oxidation of long chain fatty acids. We have previously shown significant upregulation of the isoform *Cpt1b* during COC maturation in vivo [17] here we again saw a 2-fold increase in *Cpt1b* at 10 h post-hCG though this was not statistically significant (data not shown). However, the Cpt isoforms, Cpt1a, b and c do not differ in their specificity for different activated fatty acids only in their tissue localization. Thus both our previous and current study illustrate increased Cpt1 activity indicating increased capacity for carnitine-mediated transport of activated long chain fatty acids into mitochondria during maturation in vivo. Acyl-CoA dehydrogenases catalyze the first step in FAO and those encoded by *Acadl*, *Acadm* and *Acadsb* have substrate optima of 16, 8, and 4 carbon chains, respectively [50], [51]. Expression of these isoforms indicate that the COC is capable of metabolizing fatty acids of 4–16 carbons long and increases its capacity to perform FAO during in vivo maturation. *Acaa2* codes for acetyl-CoA transferase, which catalyzes the fourth and final step in FAO, the formation of acetyl-CoA which is able to enter the TCA cycle for ATP production. *Acaa2* was significantly induced further indicating an increased capacity for the COC to perform FAO for energy production during maturation in vivo.

The significant dysregulation of 15 genes in the FAO pathway in COCs matured in vitro compared to their in vivo matured counterparts (Fig. 3) may indicate a switch in preference of fatty acids during the two different maturation conditions. This may be due to the presence of different fatty acids during IVM which are supplied via bovine serum compared to those present in the follicular fluid in which the COC matures in vivo. It would be interesting to examine whether supplementation of IVM media with fatty acids found in follicular fluid normalizes the expression level of some of the genes examined here resulting in improved oocyte developmental competence.

Acsl4 has a particular preference for arachidonic acid, a precursor for prostaglandin synthesis, and is involved in prostaglandin synthesis and release [52] with strong expression noted in steroidogenic tissues including the ovary [44]. Thus the significant down regulation of *Acsl4* in IVM COCs observed in the current study may be partly responsible for the poor PGE2 secretion observed in these COCs [53] and the poorer developmental competence of these oocytes. In support of this female *Acsl4* heterozygous null mice have reduced fertility [54] as do Ptgs2 deficient mice [55].

That *Acaa2*, which is responsible for the final step in FAO in the mouse, was significantly reduced in COCs matured in vitro indicates that FAO was deficient compared to COCs matured in vivo and this was confirmed using a functional assay. Previous studies have shown that IVM COCs have altered mitochondrial and glycolytic activity and poor oocyte developmental competence [3], [4]. The current study demonstrates that the down regulation of FAO could be responsible for the subsequent developmental deficiencies that occur in response to IVM.

The up-regulation of FAO by PPAR agonists has been reported in many different tissues and cells, however very little attention has been focused on their action within the ovary, especially the COC. Both PPARα and PPARγ mRNA were confirmed to be expressed in the COCs used in these experiments (data not shown) however it is not possible to conclude whether this includes expression specifically in the oocyte. Bezafibrate is a potent PPARα agonist shown to increase FAO in other cell lines and tissues [35], [36]. However, in the current study bezafibrate had no effect on FAO in the COC during in vitro maturation or on subsequent embryo development following IVF. Rosiglitazone has been shown to differentially modulate FAO in different cell types [56]–[58]. In the current study we show that rosiglitazone treatment of COCs during maturation in vitro dose dependently inhibited FAO despite upregulating genes involved in fatty acid activation, carnitine-mediated transport and metabolim. As a ligand for PPARγ it is possible that rosiglitazone treatment also blocks distinct effects of its endogenous fatty acid ligands. Alternatively, since rosiglitazone is known to promote fat storage in some cell types while down regulating FAO [56], rosiglitazone may activate lipid storage rather than metabolism in the COC. The observation of increased cumulus expansion in the presence of rosiglitazone suggests that cumulus matrix production is increased, perhaps due to increased carbohydrate metabolism in the face of impaired β-oxidation. Indeed, elevated flux of glucose through the hexosamine biosynthesis pathway has been shown to increase extracellular matrix substrate supply and hence COC expansion [59]. Overall, these results indicate that compared to PPARα, PPARγ is a more active regulator of COC functions during oocyte maturation.

We have previously demonstrated that in vivo administration of rosiglitazone in obese mice significantly improved oocyte developmental competence [33]. What was unclear from this study however is whether the effects of rosiglitazone on oocyte developmental competence were due to direct effects on the ovarian cells or via systemic effects such as reduced circulating insulin and lipid. The results from the current study demonstrate rosiglitazone action directly on the COC inhibited FAO and impaired subsequent embryo development supporting the conclusion that in vivo administration of rosiglitazone has indirect beneficial effects on the oocyte via its actions on the maternal systemic environment. These contradictory effects of rosiglitazone on oocyte developmental competence might also be due to differences in the availability of lipid substrate in the treated COCs. We have previously shown that mice fed high fat diet exhibit a marked increase in both oocyte and cumulus cell lipid content compared to mice fed a low fat diet [60] and perhaps in this cellular context rosiglitazone improves energy production and embryo development; but has a negative effect when lipid levels are limited.

Despite the negative effect of rosiglitazone on oocyte developmental competence we found an increase in the inner mitochondrial membrane potential (MMP) of oocytes when COCs were matured in the presence of rosiglitazone. An increase in MMP in oocytes is usually associated with improved oocyte developmental competence [61]–[63], although more recent reports have associated increased hyper-polarized mitochondria with decreased oocyte developmental competence and increased embryo fragmentation [64], [65]. Perhaps there is a threshold for a positive association between MMP and oocyte developmental competence and raising the MMP too high is detrimental due to excessive levels of reactive oxygen species (ROS). How rosiglitazone raises oocyte MMP despite significantly down-regulating FAO in the whole COC is unclear but it is likely that rosiglitazone exerts cell specific effects on cumulus cells and the oocyte, perhaps due to differential receptor expression levels between these two cell types and/or the presence of distinct co-activators.

From this and previous studies [17]–[20] it is emerging that FAO is an important metabolic pathway in determining oocyte developmental potential in vitro. We show here that FAO is induced during oocyte maturation in vivo and is deficient during IVM. The PPAR agonists bezafibrate and rosiglitazone were not effective in restoring FAO in vitro. In conclusion, further optimization of in vitro maturation conditions to normalize levels of FAO to that observed in vivo may be necessary to improve the success of IVM.

## References

[1] Edwards RG (1965) Maturation in vitro of human ovarian oocytes. Lancet 2: 926–929. 4165802 10.1016/s0140-6736(65)92903-x

[2] Gilchrist RB (2011) Recent insights into oocyte-follicle cell interactions provide opportunities for the development of new approaches to in vitro maturation. Reproduction, fertility, and development 23: 23–31. 10.1071/RD10225 21366977

[3] Krisher RL (2004) The effect of oocyte quality on development. Journal of animal science 82 Suppl: E14–23 10.2527/2004.8213_supplE14x 15471793

[4] Martino NA, Lacalandra GM, Filioli Uranio M, Ambruosi B, Caira M, et al.. (2012) Oocyte mitochondrial bioenergy potential and oxidative stress: within-/between-subject, in vivo versus in vitro maturation, and age-related variations in a sheep model. Fertility and sterility 97: 720–728 e721. 10.1016/j.fertnstert.2011.12.014 22260855

[5] Dunning KR, Lane M, Brown HM, Yeo C, Robker RL, et al (2007) Altered composition of the cumulus-oocyte complex matrix during in vitro maturation of oocytes. Hum Reprod 22: 2842–2850. 17872911 10.1093/humrep/dem277

[6] Buckett WM, Chian RC, Dean NL, Sylvestre C, Holzer HE, et al (2008) Pregnancy loss in pregnancies conceived after in vitro oocyte maturation, conventional in vitro fertilization, and intracytoplasmic sperm injection. Fertil Steril 90: 546–550. 17904128 10.1016/j.fertnstert.2007.06.107

[7] Child TJ, Phillips SJ, Abdul-Jalil AK, Gulekli B, Tan SL (2002) A comparison of in vitro maturation and in vitro fertilization for women with polycystic ovaries. Obstet Gynecol 100: 665–670. 12383531 10.1016/s0029-7844(02)02193-2

[8] Gilchrist RB, Thompson JG (2007) Oocyte maturation: emerging concepts and technologies to improve developmental potential in vitro. Theriogenology 67: 6–15. 17092551 10.1016/j.theriogenology.2006.09.027

[9] Biggers JD, Whittingham DG, Donahue RP (1967) The pattern of energy metabolism in the mouse oocyte and zygote. Proc Natl Acad Sci U S A 58: 560–567. 5233459 10.1073/pnas.58.2.560 PMC335672

[10] Thompson JG, Lane M, Gilchrist RB (2007) Metabolism of the bovine cumulus-oocyte complex and influence on subsequent developmental competence. Soc Reprod Fertil Suppl 64: 179–190. 10.5661/rdr-vi-179 17491147

[11] Downs SM (1995) The influence of glucose, cumulus cells, and metabolic coupling on ATP levels and meiotic control in the isolated mouse oocyte. Dev Biol 167: 502–512. 7875374 10.1006/dbio.1995.1044

[12] Preis KA, Seidel GEJ, Gardner DK (2007) Reduced oxygen concentration improves the developmental competence of mouse oocytes following in vitro maturation. Mol Reprod Dev 74: 893–903. 17192892 10.1002/mrd.20655

[13] Sugiura K, Eppig JJ (2005) Society for Reproductive Biology Founders' Lecture 2005. Control of metabolic cooperativity between oocytes and their companion granulosa cells by mouse oocytes. Reprod Fertil Dev 17: 667–674. 16364219 10.1071/rd05071

[14] Van Blerkom J, Davis PW, Lee J (1995) ATP content of human oocytes and developmental potential and outcome after in-vitro fertilization and embryo transfer. Hum Reprod 10: 415–424. 7769073 10.1093/oxfordjournals.humrep.a135954

[15] Downs SM, Mosey JL, Klinger J (2009) Fatty acid oxidation and meiotic resumption in mouse oocytes. Mol Reprod Dev 76: 844–853. 19455666 10.1002/mrd.21047 PMC3995453

[16] Valsangkar D, Downs SM (2013) A requirement for fatty acid oxidation in the hormone-induced meiotic maturation of mouse oocytes. Biol Reprod 89: 43. 23863407 10.1095/biolreprod.113.109058 PMC4076365

[17] Dunning KR, Cashman K, Russell DL, Thompson JG, Norman RJ, et al (2010) Beta-oxidation is essential for mouse oocyte developmental competence and early embryo development. Biol Reprod 83: 909–918. 20686180 10.1095/biolreprod.110.084145

[18] Dunning KR, Akison LK, Russell DL, Norman RJ, Robker RL (2011) Increased beta-oxidation and improved oocyte developmental competence in response to l-carnitine during ovarian in vitro follicle development in mice. Biol Reprod 85: 548–555. 21613630 10.1095/biolreprod.110.090415

[19] McKeegan PJ, Sturmey RG (2011) The role of fatty acids in oocyte and early embryo development. Reprod Fertil Dev 24: 59–67. 22394718 10.1071/RD11907

[20] Hashimoto S (2009) Application of in vitro maturation to assisted reproductive technology. J Reprod Dev 55: 1–10. 19276618 10.1262/jrd.20127

[21] Somfai T, Kaneda M, Akagi S, Watanabe S, Haraguchi S, et al (2011) Enhancement of lipid metabolism with L-carnitine during in vitro maturation improves nuclear maturation and cleavage ability of follicular porcine oocytes. Reprod Fertil Dev 23: 912–920. 21871210 10.1071/RD10339

[22] Takahashi T, Inaba Y, Somfai T, Kaneda M, Geshi M, et al (2013) Supplementation of culture medium with L-carnitine improves development and cryotolerance of bovine embryos produced in vitro. Reprod Fertil Dev 25: 589–599. 22954232 10.1071/RD11262

[23] Wu GQ, Jia BY, Li JJ, Fu XW, Zhou GB, et al (2011) L-carnitine enhances oocyte maturation and development of parthenogenetic embryos in pigs. Theriogenology 76: 785–793. 21705056 10.1016/j.theriogenology.2011.04.011

[24] You J, Lee J, Hyun SH, Lee E (2012) L-carnitine treatment during oocyte maturation improves in vitro development of cloned pig embryos by influencing intracellular glutathione synthesis and embryonic gene expression. Theriogenology 78: 235–243. 22578613 10.1016/j.theriogenology.2012.02.027

[25] Ferguson EM, Leese HJ (2006) A potential role for triglyceride as an energy source during bovine oocyte maturation and early embryo development. Mol Reprod Dev 73: 1195–1201. 16804881 10.1002/mrd.20494

[26] Cabrero A, Cubero M, Llaverias G, Jove M, Planavila A, et al (2003) Differential effects of peroxisome proliferator-activated receptor activators on the mRNA levels of genes involved in lipid metabolism in primary human monocyte-derived macrophages. Metabolism: clinical and experimental 52: 652–657. 12759900 10.1053/meta.2003.50100

[27] Sprecher DL, Massien C, Pearce G, Billin AN, Perlstein I, et al (2007) Triglyceride: high-density lipoprotein cholesterol effects in healthy subjects administered a peroxisome proliferator activated receptor delta agonist. Arteriosclerosis, thrombosis, and vascular biology 27: 359–365. 10.1161/01.ATV.0000252790.70572.0c 17110604

[28] Froment P, Gizard F, Defever D, Staels B, Dupont J, et al (2006) Peroxisome proliferator-activated receptors in reproductive tissues: from gametogenesis to parturition. The Journal of endocrinology 189: 199–209. 16648288 10.1677/joe.1.06667

[29] Issemann I, Green S (1990) Activation of a member of the steroid hormone receptor superfamily by peroxisome proliferators. Nature 347: 645–650. 2129546 10.1038/347645a0

[30] Dreyer C, Krey G, Keller H, Givel F, Helftenbein G, et al (1992) Control of the peroxisomal beta-oxidation pathway by a novel family of nuclear hormone receptors. Cell 68: 879–887. 1312391 10.1016/0092-8674(92)90031-7

[31] Vamecq J, Latruffe N (1999) Medical significance of peroxisome proliferator-activated receptors. Lancet 354: 141–148. 10408502 10.1016/S0140-6736(98)10364-1

[32] Kliewer SA, Umesono K, Noonan DJ, Heyman RA, Evans RM (1992) Convergence of 9-cis retinoic acid and peroxisome proliferator signalling pathways through heterodimer formation of their receptors. Nature 358: 771–774. 1324435 10.1038/358771a0 PMC6159883

[33] Minge CE, Bennett BD, Norman RJ, Robker RL (2008) Peroxisome proliferator-activated receptor-gamma agonist rosiglitazone reverses the adverse effects of diet-induced obesity on oocyte quality. Endocrinology 149: 2646–2656. 18276752 10.1210/en.2007-1570

[34] Pfaffl MW, Tichopad A, Prgomet C, Neuvians TP (2004) Determination of stable housekeeping genes, differentially regulated target genes and sample integrity: BestKeeper – Excel-based tool using pair-wise correlations. Biotechnol Lett 26: 509–515. 15127793 10.1023/b:bile.0000019559.84305.47

[35] Djouadi F, Bonnefont JP, Thuillier L, Droin V, Khadom N, et al (2003) Correction of fatty acid oxidation in carnitine palmitoyl transferase 2-deficient cultured skin fibroblasts by bezafibrate. Pediatric research 54: 446–451. 12840153 10.1203/01.PDR.0000083001.91588.BB

[36] Cabrero A, Alegret M, Sanchez RM, Adzet T, Laguna JC, et al (2001) Bezafibrate reduces mRNA levels of adipocyte markers and increases fatty acid oxidation in primary culture of adipocytes. Diabetes 50: 1883–1890. 11473052 10.2337/diabetes.50.8.1883

[37] Djouadi F, Aubey F, Schlemmer D, Bastin J (2005) Peroxisome proliferator activated receptor delta (PPARdelta) agonist but not PPARalpha corrects carnitine palmitoyl transferase 2 deficiency in human muscle cells. The Journal of clinical endocrinology and metabolism 90: 1791–1797. 15613406 10.1210/jc.2004-1936

[38] Vandewalle B, Moerman E, Lefebvre B, Defrance F, Gmyr V, et al (2008) PPARgamma-dependent and -independent effects of rosiglitazone on lipotoxic human pancreatic islets. Biochemical and biophysical research communications 366: 1096–1101. 18155663 10.1016/j.bbrc.2007.12.088

[39] Wang P, Renes J, Bouwman F, Bunschoten A, Mariman E, et al (2007) Absence of an adipogenic effect of rosiglitazone on mature 3T3-L1 adipocytes: increase of lipid catabolism and reduction of adipokine expression. Diabetologia 50: 654–665. 17245590 10.1007/s00125-006-0565-0 PMC1914285

[40] Djaouti L, Jourdan T, Demizieux L, Chevrot M, Gresti J, et al (2010) Different effects of pioglitazone and rosiglitazone on lipid metabolism in mouse cultured liver explants. Diabetes/metabolism research and reviews 26: 297–305. 20503262 10.1002/dmrr.1081

[41] Vanderhyden BC, Caron PJ, Buccione R, Eppig JJ (1990) Developmental pattern of the secretion of cumulus expansion-enabling factor by mouse oocytes and the role of oocytes in promoting granulosa cell differentiation. Dev Biol 140: 307–317. 2115479 10.1016/0012-1606(90)90081-s

[42] Pei Z, Jia Z, Watkins PA (2006) The second member of the human and murine bubblegum family is a testis- and brainstem-specific acyl-CoA synthetase. J Biol Chem 281: 6632–6641. 16371355 10.1074/jbc.M511558200

[43] Iijima H, Fujino T, Minekura H, Suzuki H, Kang MJ, et al (1996) Biochemical studies of two rat acyl-CoA synthetases, ACS1 and ACS2. Eur J Biochem 242: 186–190. 8973631 10.1111/j.1432-1033.1996.0186r.x

[44] Kang MJ, Fujino T, Sasano H, Minekura H, Yabuki N, et al (1997) A novel arachidonate-preferring acyl-CoA synthetase is present in steroidogenic cells of the rat adrenal, ovary, and testis. Proc Natl Acad Sci U S A 94: 2880–2884. 9096315 10.1073/pnas.94.7.2880 PMC20291

[45] Oikawa E, Iijima H, Suzuki T, Sasano H, Sato H, et al (1998) A novel acyl-CoA synthetase, ACS5, expressed in intestinal epithelial cells and proliferating preadipocytes. J Biochem 124: 679–685. 9722683 10.1093/oxfordjournals.jbchem.a022165

[46] Homa ST, Brown CA (1992) Changes in linoleic acid during follicular development and inhibition of spontaneous breakdown of germinal vesicles in cumulus-free bovine oocytes. J Reprod Fertil 94: 153–160. 1552477 10.1530/jrf.0.0940153

[47] Jungheim ES, Macones GA, Odem RR, Patterson BW, Lanzendorf SE, et al (2011) Associations between free fatty acids, cumulus oocyte complex morphology and ovarian function during in vitro fertilization. Fertil Steril 95: 1970–1974. 21353671 10.1016/j.fertnstert.2011.01.154 PMC3080431

[48] Tsujii H, Khandoker MAMY, Hamano K (2001) Lipid in mammalian embryo development. J Mamm Ova Res 18: 73–80.

[49] O'Gorman A, Wallace M, Cottell E, Gibney MJ, McAuliffe FM, et al.. (2013) Metabolic profiling of human follicular fluid identifies potential biomarkers of oocyte developmental competence. Reproduction. 10.1530/REP-13-0184 23886995

[50] Finocchiaro G, Ito M, Ikeda Y, Tanaka K (1988) Molecular cloning and nucleotide sequence of cDNAs encoding the alpha-subunit of human electron transfer flavoprotein. J Biol Chem 263: 15773–15780. 3170610

[51] He M, Burghardt TP, Vockley J (2003) A novel approach to the characterization of substrate specificity in short/branched chain Acyl-CoA dehydrogenase. J Biol Chem 278: 37974–37986. 12855692 10.1074/jbc.M306882200

[52] Golej DL, Askari B, Kramer F, Barnhart S, Vivekanandan-Giri A, et al (2011) Long-chain acyl-CoA synthetase 4 modulates prostaglandin E(2) release from human arterial smooth muscle cells. J Lipid Res 52: 782–793. 21242590 10.1194/jlr.M013292 PMC3053208

[53] Dunning KR, Watson LN, Sharkey DJ, Brown HM, Norman RJ, et al (2012) Molecular filtration properties of the mouse expanded cumulus matrix: controlled supply of metabolites and extracellular signals to cumulus cells and the oocyte. Biol Reprod 87: 89. 22837478 10.1095/biolreprod.111.096271

[54] Cho YY, Kang MJ, Sone H, Suzuki T, Abe M, et al (2001) Abnormal uterus with polycysts, accumulation of uterine prostaglandins, and reduced fertility in mice heterozygous for acyl-CoA synthetase 4 deficiency. Biochem Biophys Res Commun 284: 993–997. 11409893 10.1006/bbrc.2001.5065

[55] Lim H, Paria BC, Das SK, Dinchuk JE, Langenbach R, et al (1997) Multiple female reproductive failures in cyclooxygenase 2-deficient mice. Cell 91: 197–208. 9346237 10.1016/s0092-8674(00)80402-x

[56] Roberts LD, Murray AJ, Menassa D, Ashmore T, Nicholls AW, et al (2011) The contrasting roles of PPARdelta and PPARgamma in regulating the metabolic switch between oxidation and storage of fats in white adipose tissue. Genome Biol 12: R75. 21843327 10.1186/gb-2011-12-8-r75 PMC3245615

[57] Wilmsen HM, Ciaraldi TP, Carter L, Reehman N, Mudaliar SR, et al (2003) Thiazolidinediones upregulate impaired fatty acid uptake in skeletal muscle of type 2 diabetic subjects. Am J Physiol Endocrinol Metab 285: E354–362. 12700163 10.1152/ajpendo.00491.2001

[58] Benton CR, Holloway GP, Campbell SE, Yoshida Y, Tandon NN, et al (2008) Rosiglitazone increases fatty acid oxidation and fatty acid translocase (FAT/CD36) but not carnitine palmitoyltransferase I in rat muscle mitochondria. J Physiol 586: 1755–1766. 18238811 10.1113/jphysiol.2007.146563 PMC2375706

[59] Sutton-McDowall ML, Gilchrist RB, Thompson JG (2004) Cumulus expansion and glucose utilisation by bovine cumulus-oocyte complexes during in vitro maturation: the influence of glucosamine and follicle-stimulating hormone. Reproduction 128: 313–319. 15333782 10.1530/rep.1.00225

[60] Wu LL, Dunning KR, Yang X, Russell DL, Lane M, et al (2010) High-fat diet causes lipotoxicity responses in cumulus-oocyte complexes and decreased fertilization rates. Endocrinology 151: 5438–5445. 20861227 10.1210/en.2010-0551

[61] Van Blerkom J, Davis P (2006) High-polarized (Delta Psi m(HIGH)) mitochondria are spatially polarized in human oocytes and early embryos in stable subplasmalemmal domains: developmental significance and the concept of vanguard mitochondria. Reprod Biomed Online 13: 246–254. 16895640 10.1016/s1472-6483(10)60622-0

[62] Van Blerkom J, Davis P, Alexander S (2003) Inner mitochondrial membrane potential (DeltaPsim), cytoplasmic ATP content and free Ca2+ levels in metaphase II mouse oocytes. Hum Reprod 18: 2429–2440. 14585897 10.1093/humrep/deg466

[63] Van Blerkom J, Davis P, Mathwig V, Alexander S (2002) Domains of high-polarized and low-polarized mitochondria may occur in mouse and human oocytes and early embryos. Hum Reprod 17: 393–406. 11821285 10.1093/humrep/17.2.393

[64] Acton BM, Jurisicova A, Jurisica I, Casper RF (2004) Alterations in mitochondrial membrane potential during preimplantation stages of mouse and human embryo development. Mol Hum Reprod 10: 23–32. 14665703 10.1093/molehr/gah004

[65] Zander-Fox D, Cashman KS, Lane M (2013) The presence of 1 mM glycine in vitrification solutions protects oocyte mitochondrial homeostasis and improves blastocyst development. J Assist Reprod Genet 30: 107–116. 23248076 10.1007/s10815-012-9898-4 PMC3553346

